# The Effect of Dexamethasone on Neuroinflammation and Cerebral Edema in Rats With Traumatic Brain Injury Combined With Seawater Drowning

**DOI:** 10.7759/cureus.55309

**Published:** 2024-03-01

**Authors:** Zihuan Zeng, Liangfeng Wei, Hao Zhang, Weiqiang Chen, Shousen Wang

**Affiliations:** 1 Department of Neurosurgery, Fuzhou 900th Hospital, Fuzong Clinical Medical College of Fujian Medical University, Fuzhou, CHN; 2 Department of Neurosurgery, Fuzhou 900th Hospital, Fujian Medical University, Fuzhou, CHN; 3 Department of Neurosurgery, The First Affiliated Hospital, Shantou University Medical College, Shantou, CHN

**Keywords:** rat, dexamethasone, diffuse axonal injury, seawater drowning, traumatic brain injury

## Abstract

Objective: To investigate the effect and mechanism of dexamethasone (DX) on axonal injury after traumatic brain injury (TBI) combined with seawater drowning (SWD) in rats.

Methods: To gain an in-depth understanding of TBI + SWD in rats, we established the compound injury model of rats by the Marmarou method and intratracheal pumping of seawater to simulate the pathological conditions. Rats in the DX group received intraperitoneal injections of DX (1 mg/kg) immediately after injury, and rats in the sham group and TBI + SWD group received intraperitoneal injections of the same amount of normal saline.

Results: Hematoxylin-eosin (HE) showed that DX improved matrix looseness, cell swelling, and nuclear condensation 168 hours after injury. Immunohistochemistry (IHC) staining showed that the protein expression of AQP4 was decreased in the DX group compared with the TBI + SWD group from 12 hours to 168 hours after injury. DX decreased the modified neurological severity score (mNSS) significantly at 24 hours and 168 hours after injury (P < 0.05). At 72 h and 168 h after injury, DX significantly lowered the expressions of IL-8 and TNF-α (P < 0.05).

Conclusion: DX may play a neuroprotective role by reducing cerebral edema and inflammatory response after TBI + SWD injury in rats.

## Introduction

Traumatic brain injuries (TBIs) are a serious public health concern worldwide, with recent estimates reporting that 10-60 million new cases occur annually [1,2]. TBI can be classified into primary and secondary brain injuries. Secondary brain injury is the focus of medical treatment [2]. TBI can occur when people are developing and utilizing marine resources. Seawater will be accidentally inhaled into the lungs of TBI patients who are unconscious before falling into the water, which causes seawater drowning (SWD). This compound injury is difficult to treat [3,4]. Based on the complex pathophysiological process of TBI + SWD, high-quality brain protectants should have multiple neuroprotective effects. Multi-effect neuroprotective drugs have been the mainstream of TBI therapy.

Pro-inﬂammatory cytokines, such as interleukin-8 (IL-8) and tumor necrosis factor-α (TNFα), drive inﬂammatory activation and promote cellular inﬂammation. Suppressing excessive inﬂammation following injury is a solid target for improving recovery [5]. Dexamethasone (DX) has demonstrated signiﬁcant reduction in early pro-inﬂammatory, cytokine expression when used in high-dose systemic administration. The use of DX for the treatment of head trauma spans four decades [6]. However, the long-term, high-dose administration of DX is also fraught with serious side effects, such as diabetes and glaucoma. Additionally, the international, randomized, double-blind clinical trial Corticosteroid Randomisation After Significant Head Injury (known as CRASH) reported increased mortality [7] from high-dose synthetic glucocorticoid (GC) administration in the treatment of moderate-to-severe TBI. Other studies have observed no significant beneﬁts of high-dose GC treatment [8,9]. These have considerably contraindicated the use of DX in recent clinical guidelines [2]. However, several studies have reported that low-dose DX can reduce secondary injury and improve functional recovery [10-12]. DX hydrogels covering the surface of the brain reduce the inﬂammatory response, apoptosis, and lesion volume compared to untreated animals at 14 days after injury [13]. DX prodrug-treated TBI mice demonstrated that their neuronal protection has benefits after TBI [14].

In this study, the rat model with closed TBI and SWD [15] is established by using the Marmarou model [16] and pumping seawater into the trachea. On this basis, we extend our investigation of the therapeutic efficacy of DX to a longer period (168 h). Cognitive functional assessment is evaluated by modified neurological severity score (mNSS) at 168 h. Upon completion of the cognitive assessment, tissue samples were collected for the histological analysis of the effect of DX on neuroinflammation, lesion volume, and edema.

## Materials and methods

Experimental animals and materials

All animal experiments were approved by the 900th Hospital Ethics Committee (Fuzhou, China) and conducted under its strict supervision. Healthy male specific-pathogen-free-grade Sprague-Dawley (SD) rats weighing 350 ± 20 g were purchased from Shanghai SLAC Laboratory Animal Co. Ltd. Sea water was artificially prepared according to the formulation from the Third Institute of Oceanography. Other purchases and preparations for this study were DX (purity ≧ 98%, Shanghai Macklin Biochemical Technology Co. Ltd., China); Marmarou device of traumatic brain injury (Imitation); rabbit polyclonal antibody to Aquaporin 4 (AQP4) (Abcam, UK); and mouse anti-TNF-α enzyme-linked immunosorbent assay (ELISA) kit (96T) (Abcam, UK).

Grouping and processing of experimental animals

According to the random number table, 78 SD male rats were randomly divided into the sham group (n = 14), the TBI + SWD group (n = 32), and the DX group (n = 32). The TBI + SWD group and the DX group were divided into four subgroups according to the time points 12 h, 24 h, 72 h, and 168 h after the TBI + SWD model. In each group, there were eight rats in the 24-hour subgroup and six rats in the 168-hour subgroup. In the TBI + SWD group and the DX group, there were eight rats in subgroups from each time point. In the TBI + SWD group and the DX group, six rats were randomly selected from subgroups at different time points for TNF-α and IL-8 protein expression tests, and the remaining two rats were used for hematoxylin-eosin (HE) and immunohistochemistry (IHC) testing. All the rats in the 168-hour subgroup were tested for mNSS. All rats were sacrificed at a scheduled time.

Establishment of TBI + SWD rat model

The TBI rat model was prepared with the following procedures. According to the Marmarou brain injury model, the TBI model was first established, followed by SWD [15]. After a successful model was established, we sutured the skin incision, and the observation started after the rats were fully awake.

Treatment

By intraperitoneal injection of drugs, all DX-treated rats were treated with 1 mL of DX (1 mg/kg) immediately after injury, every 24 hours injected until the rats were sacrificed. The sham group was given an equal volume of normal saline. 

mNSS

mNSS [17] was employed in this study for the three groups of 168-hour subgroups. Rats were scored after 12 h, 24 h, 72 h, and 168 h (by a single-blind member). All rats were scored normally before surgery (0 points). If the score was not normal, the rat was excluded, and new rats were added randomly.

Determination of the brain water content

The whole brain was decapitated and placed on ice at the scheduled time. About 0.5 g of brain tissue was taken in the wounded area, and placed on a neat glass slide, and the wet weight was measured by a precision electronic analytical balance. The slide with tissue was dried to constant weight, and its dry weight was measured twice. Brain water content was calculated according to the equation: brain water content (%) = (wet weight - dry weight)/wet weight × 100%.

HE staining

Rat whole-brain tissue was perfused with normal saline and 4% paraformaldehyde, followed by being fixed in 4% paraformaldehyde for 24 hours. After being dehydrated, transparently treated, embedded in paraffin, and cut into 4 μm thick sections from the head to the end, HE staining was observed.

IHC

After being paraffin-embedded, sectioned, dewaxed, and hydrated, AQP4 was repaired by ethylenediaminetetraacetic acid (pH = 9.0) at the same heating condition. According to the instructions of the immunohistochemical kit, the test material was incubated at room temperature for 20 minutes and then added to the enzyme avidin peroxidase. The test sample was colored by diaminobenzidine, slightly re-dyed by hematoxylin, dehydrated by gradient alcohol, made transparent by xylene, and finally sealed with neutral gum. The expression of AQP4 in rat brain tissue was observed under a microscope. For the immunohistochemical staining results, five 400x areas were chosen on each slice and each field of vision was analyzed for dyeing strength and percentage of positive cells. The analysis was carried out according to the standard reference literature. When combining the dyeing strength results and positive cell percentage for IHC result determination, the immunohistochemical assay results were divided into negative (-), weak positive (+), moderate positive (++), and strong positive (+++). The positive expressions of AQP4 in this test were tan.

ELISA

After cell lysis and centrifugation of rat hippocampal tissue, supernatant was taken, and precooled normal saline was added at a mass-to-volume ratio of 1:9 to prepare brain tissue homogenate with a volume fraction of 10%. The prepared sample and standard substance of 100 μL were added to the reaction well and incubated for 2 h at 37℃. The liquid in the well was discarded, and the test solution A was added to each well, which was shaken well and incubated for another 60 min at 37℃. The liquid in the well was then discarded, and the washing solution was added to the plate five times. After being incubated at 37℃ in the dark for 30 min, the absorbance (A) of each well was measured at 450 nm wavelength by adding 50 μL termination solution. The corresponding concentration was calculated, and the concentration multiplied by dilution was the expression level of IL-8 and TNF-α in the hippocampus.

Statistical analysis

Statistical analysis was performed using Statistical Package for the Social Sciences 22.0 software (known as SPSS; Armonk, NY: IBM Corp). Experimental data were expressed as the mean ± standard deviation. Normal tests and homogeneity tests of variance were carried out. We used a two-factor analysis of variance, and then the analysis of variance was performed on the two factors of grouping and time. The least significant difference test method was used for further comparison between the two factors. If the homogeneity of the variance was not met, the Kruskal-Wallis H method was used for the rank-sum test. The significant level (P) was set at 0.05.

## Results

mNSS of rats

**Figure 1:**
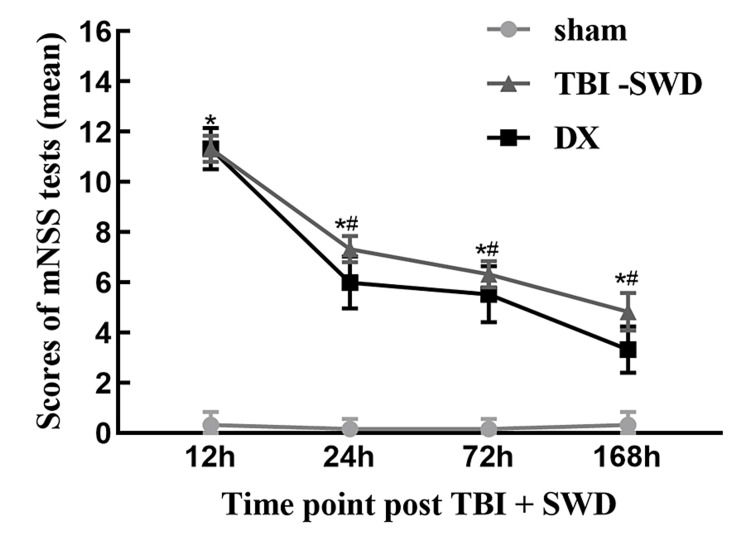
The mNSS of rats Compared with the sham group, the mNSS of the TBI + SWD group and the DX group were increased. *P < 0.05 vs. sham group; compared with the TBI + SWD group, the mNSS in the DX group decreased from 12 h to 168 h after injury. #P < 0.05 vs. TBI + SWD group. mNSS, modified neurological severity score; TBI, traumatic brain injury; SWD, seawater drowning.

The mNSS of rats in both the TBI + SWD group and the DX group decreased with time from 12 h after injury. Compared with the sham group, the mNSS of both the TBI + SWD and DX groups increased with a statistically significant difference (P < 0.05). Compared with the TBI + SWD group, the mNSS in the DX group decreased from 24 h to 168 h after injury (P < 0.05) (Figure 1).

Brain water content

**Figure 2:**
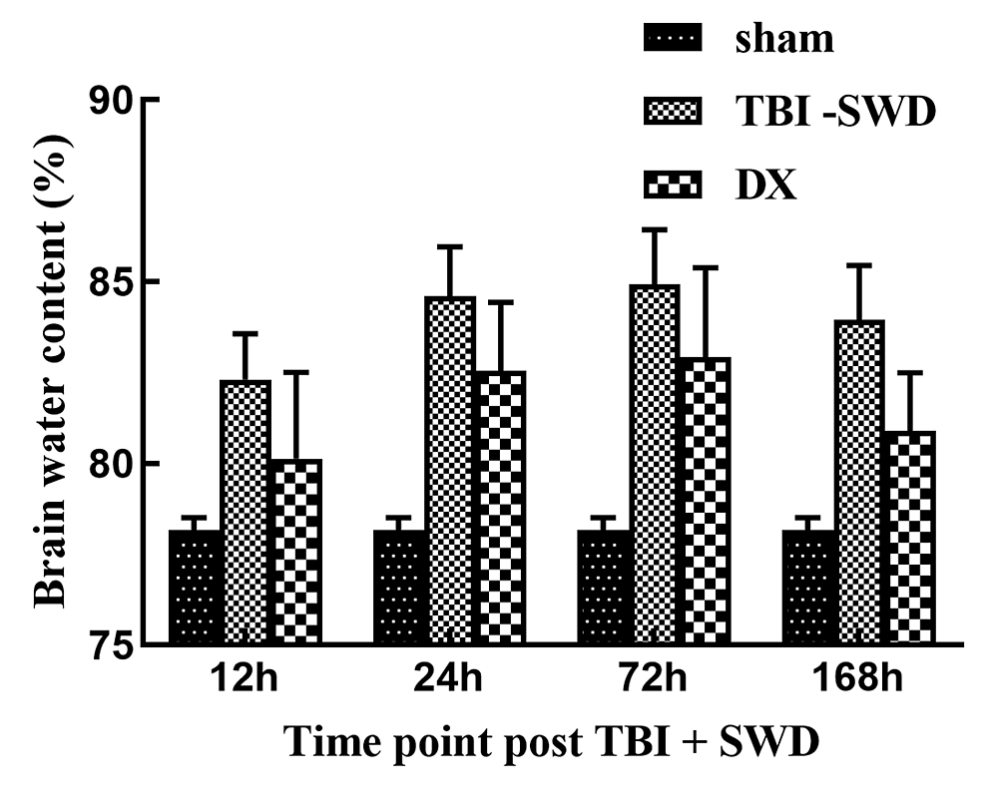
Brain water content of rats The brain water content in the DX group was smaller than in the TBI + SWD group at 72 h and 168 h after injury. P > 0.05. DX, dexamethasone; TBI, traumatic brain injury; SWD, seawater drowning.

Compared with the sham group, brain water content in the TBI + SWD group (12 h, 24 h, 72 h, 168 h) and DX group (12 h, 24 h, 72 h, 168 h) was significantly increased (P < 0.05). Compared with the TBI + SWD group, the water content of brain tissue in the DX group (72 h and 168 h) was smaller than that in the TBI + SWD group at 72 h and 168 h after injury (P > 0.05) (Figure 2).

HE staining

In the sham group, the morphology and structure of neurons and glial cells were normal. In the TBI + SWD group, at 12 h and 24 h after injury, the stroma was loose and looked like a sponge, the pericellular space and perivascular space were significantly increased, the astrocyte swelling was obvious, and the blood vessel dilatation and congestion were observed in the brain parenchyma. At 72 h after injury, the encephaledema was still obvious, and the vasodilatation and congestion in the brain parenchyma were more obvious, with red blood cell spillage. At 168 h after injury, there was obvious encephaledema, hyperchromatic nuclei, and pyknosis.

**Figure 3:**
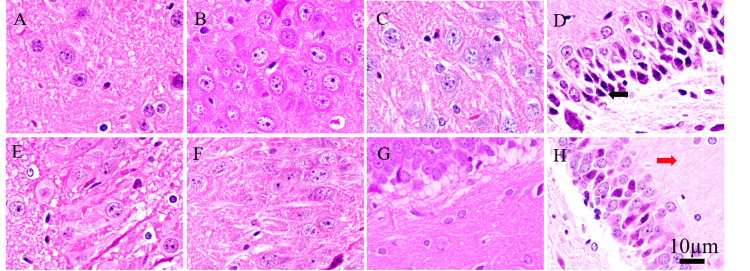
Results of HE staining of hippocampus in brain tissue for the sham group, TBI + SWD group, and DX group (A-D) 12-hour, 24-hour, 72-hour, and 168-hour subgroups of TBI + SWD, respectively; (E-H) 12-hour, 24-hour, 72-hour, and 168-hour subgroups of the DX group, respectively. Compared with the TBI + SWD group, tissue edema was reduced, cell morphology was relatively swollen, and nuclear condensation was mild. The black arrows indicate the deviated nuclear condensation. The red arrow indicates matrix looseness. Scar bar = 10 µm in A-H. HE, hematoxylin-eosin; DX, dexamethasone; TBI, traumatic brain injury; SWD, seawater drowning.

In the DX group, at 12 h after injury, the cell bodies of neurons and glial cells were similar to those of the TBI + SWD group. At 24 h and 72 h after injury, the tissue edema was reduced compared with that of the TBI + SWD group, and the cell morphology was normal with mild nuclear consolidation. But at 168 h after injury, compared with the TBI + SWD group, the loose stroma and the nuclear consolidation were reduced slightly (Figure 3).

IHC staining of AQP4

**Figure 4:**
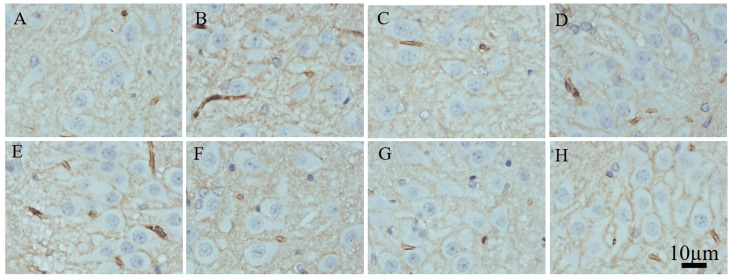
Results of IHC staining of AQP4 in the hippocampus for the TBI + SWD group and the DX group (A-D) 12-hour, 24-hour, 72-hour, and 168-hour subgroups of TBI + SWD group, respectively; (E-H) 12-hour, 24-hour, 72-hour, and 168-hour subgroups of DX group, respectively. Compared with the TBI + SWD group, the protein expressions of AQP4 were decreased in the DX group. Scale bar = 10 µm in A-H. IHC, immunohistochemistry; AQP4, aquaporin 4; DX, dexamethasone; TBI, traumatic brain injury; SWD, seawater drowning.

In the sham group, no significant expression of AQP4 was observed in the cytoplasm and axons of neurons. In the TBI + SWD group, at 12 h after injury, the subserosal and axon of hippocampal neurons showed brown staining, corresponding to positive expression (+~++) of AQP4. At 24 h, 72 h, and 168 h after injury, the positive expression of AQP4 in the subserosal and axons of hippocampal neurons was positive (++~++). In the DX group, weak positive expression (+) was observed in the cytoplasm and axons of neurons at 12 h, 24 h, 72 h, and 168 h after injury. The expression in the cytoplasm and axons of neurons was still weakly positive 168 h after injury (Figure 4).

ELISA of TNF-α, IL-8

**Figure 5:**
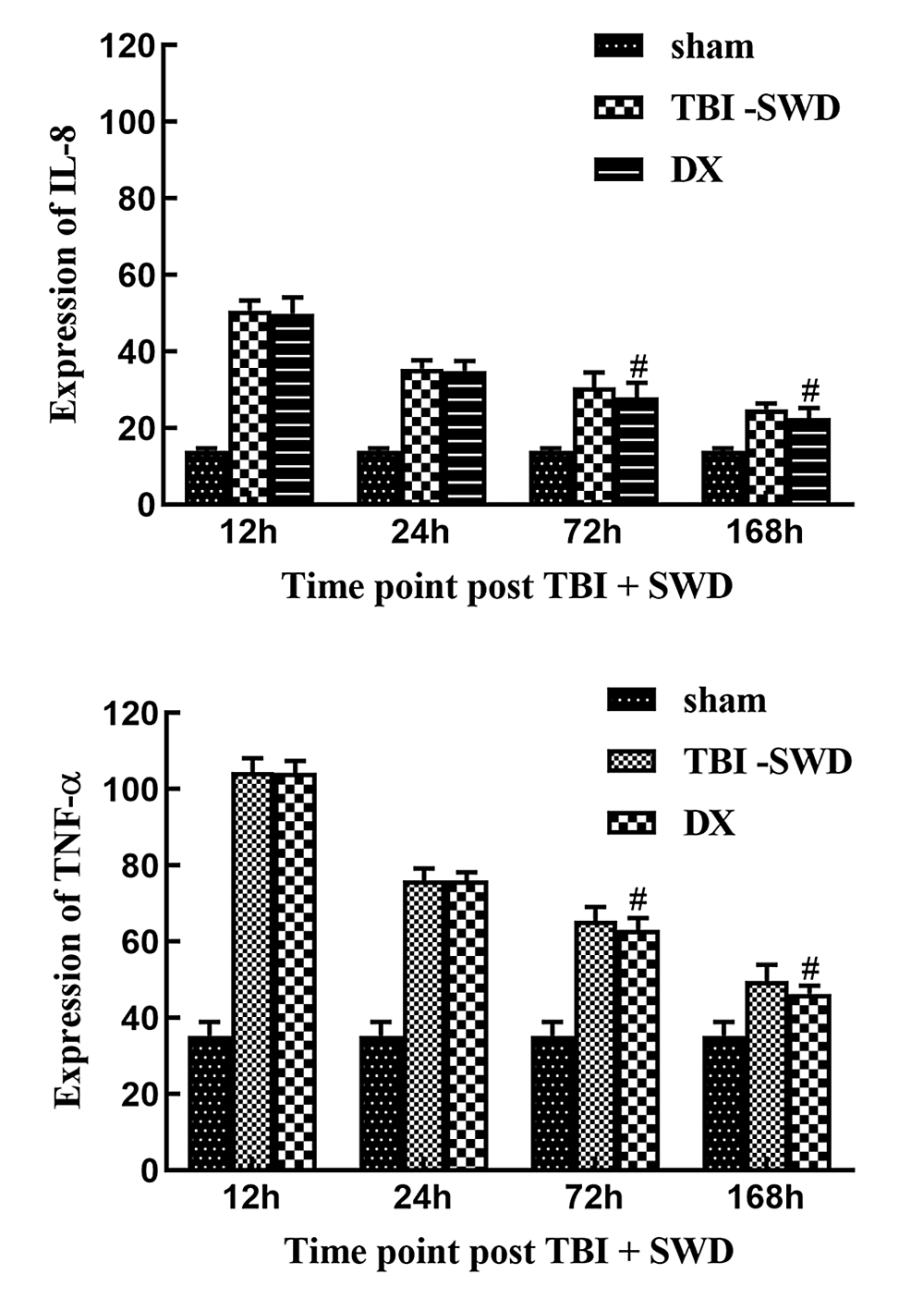
The expression of IL-8 and TNF-α in rats Compared with the TBI + SWD group, the expression of IL-8 and TNF-α in the DX group was significantly lower at 72 h and 168 h. #P < 0.05 vs. TBI + SWD group. IL-8, interleukin 8; TNF-α, tumor necrosis factor α; DX, dexamethasone; TBI, traumatic brain injury; SWD, seawater drowning.

Compared with the sham group, TNF-α and IL-8 levels in the brain tissues of the rat in the TBI + SWD group significantly increased, reaching a peak at 12 h, then slowly declined, and did not return to normal levels at 168 h after injury (P < 0.05). Compared with the TBI + SWD group, the expression of TNF-α and IL-8 at 72 h and 168 h in the DX group was significantly decreased (P < 0.05) (Figure 5).

## Discussion

TBI is one of the major causes of incapacity and mortality in the United States [18]. The brain dysfunction caused by the outside force triggers neuroinflammation and degeneration. TBI + SWD is a kind of compound injury with rapid occurrence and a critical condition [2]. The primary injuries that result from TBI subsequently initiate a chronic secondary injury phase that is characterized by widespread neuroinﬂammation and excitotoxicity. Progressive neuroinﬂammation contributes to secondary neuronal cell death after TBI and resident microglia become activated by the release of chemokines and apoptotic elements following injury [19]. Vascular damage from the primary injury allows the entry of peripheral immune cells [20,21]. Activated microglia, and to a greater degree, peripheral macrophages [22], secrete proinflammatory cytokines, such as IL-1β, IL-8, and TNF-α, leading to progressive and widespread damage to the nervous system [19].

Because of its high mortality rate, the prognosis is extremely poor if no timely and effective treatment plan is obtained. In addition, because of its complicated pathophysiological mechanism after injury, it is particularly important to select appropriate therapeutic drugs to intervene and improve prognosis. In this study, the TBI + SWD mouse model was constructed using the modified Marmarou model and the micropump intratracheal pumping method. Previous studies have confirmed that the Marmarou model is mainly composed of diffuse axonal injury, diffuse axonal fracture, and axial pulp transport failure [23]. In our previous study, we observed similar effects of low doses of DX delivery [24]. We demonstrated that delivered low-dose DX (1 mg/kg) can reduce lesion volume, neuroinﬂammatory response, and mNSS recovery in the 168-hour TBI + SWD model.

TBI and SWD can cause severe damage to the blood-brain barrier, which can lead to brain edema and cell death. While GCs have been extensively used to reduce cerebral edema in TBI patients, the clinical outcome has been controversial [25].

Early DX and inflammatory factors may enter the brain tissue upon damage to the blood-brain barrier [26]. In this study, the post-traumatic expressions of IL-8 and TNF-α in the rat hippocampal tissues were positive after treatment with DX intervention but were significantly decreased compared to the TBI + SWD group at 72 h and 168 h. mNSS and pathology can reflect the overall level of the mice’s brain tissues. The results showed that the mNSS in both the TBI + SWD and DX groups were gradually damaged after injury, and the nerve function gradually recovered but did not reach the normal level within 168 h. However, the mNSS was significantly reduced after DX intervention. In terms of pathology, the tissue structure of rats in the TBI + SWD group seriously changed 24 hours after injury. The stroma was loose, the intercellular space was significantly enlarged, the astrocytes were swollen, and the nuclei were hyperchromatic and contracted. The loose matrix, interstitial space, and cell swelling in the DX group were all improved. These findings indicated that DX can improve the neurological function and pathological changes of the hippocampus in the TBI + SWD rat.

Our study has several limitations. We included only a small number of treatment groups with different doses of DX. It is perhaps that the inclusion of more samples would have discovered further correlations. All experiments were conducted on rodents. Ulteriorly investigation with large animals is essential.

## Conclusions

We demonstrated that the sustained delivery of DX (1 mg/kg) reduced edema and inflammatory response after TBI + SWD injury in rats, and played a neuroprotective role. In the early stage of acute brain injury, the blood-brain barrier is damaged, and DX can enter the central nervous system to play an anti-inflammatory role and repair the damaged nerve.

## References

[1] Dewan MC, Rattani A, Gupta S (2018). Estimating the global incidence of traumatic brain injury. J Neurosurg.

[2] Hawryluk GW, Rubiano AM, Totten AM (2020). Guidelines for the management of severe traumatic brain injury: 2020 Update of the decompressive craniectomy recommendations. Neurosurgery.

[3] Layon AJ, Modell JH (2009). Drowning: Update 2009. Anesthesiology.

[4] Hu PJ, Pittet JF, Kerby JD, Bosarge PL, Wagener BM (2017). Acute brain trauma, lung injury, and pneumonia: More than just altered mental status and decreased airway protection. Am J Physiol Lung Cell Mol Physiol.

[5] Pearn ML, Niesman IR, Egawa J (2017). Pathophysiology associated with traumatic brain injury: Current treatments and potential novel therapeutics. Cell Mol Neurobiol.

[6] Hall ED (1985). High-dose glucocorticoid treatment improves neurological recovery in head-injured mice. J Neurosurg.

[7] Edwards P, Arango M, Balica L (2005). Final results of MRC CRASH, a randomized placebo-controlled trial of intravenous corticosteroid in adults with head injury outcomes at 6 months. Lancet.

[8] Din B, Wang X, Shi Y, Li Y (2015). Long-term effect of high-dose dexamethasone with or without low-dose dexamethasone maintenance in untreated immune thrombocytopenia. Acta Haematol.

[9] Bratton SL, Chestnut RM, Ghajar J (2007). Guidelines for the management of severe traumatic brain injury. XV. Steroids. J Neurotrauma.

[10] Holmin S, Mathiesen T (1996). Dexamethasone and colchicine reduce inflammation and delayed oedema following experimental brain contusion. Acta Neurochir (Wien).

[11] Zhang Z, Zhang Z, Artelt M, Burnet M, Schluesener HJ (2007). Dexamethasone attenuates early expression of three molecules associated with microglia/macrophages activation following rat traumatic brain injury. Acta Neuropathol.

[12] Campolo M, Ahmad A, Crupi R, Impellizzeri D, Morabito R, Esposito E, Cuzzocrea S (2013). Combination therapy with melatonin and dexamethasone in a mouse model of traumatic brain injury. J Endocrinol.

[13] Macks C, Jeong D, Bae S, Webb K, Lee JS (2022). Dexamethasone-loaded hydrogels improve motor and cognitive functions in a rat mild traumatic brain injury model. Int J Mol Sci.

[14] Wei X, Zhao G, Jia Z (2022). Macromolecular dexamethasone prodrug ameliorates neuroinflammation and prevents bone loss associated with traumatic brain injury. Mol Pharm.

[15] Zeng Z, Zhang H, Wu J, Wei L, Chen W, Wang S (2021). Effects and mechanism of mouse nerve growth factor on diffuse brain injury in rats with traumatic brain injury combined with seawater drowning. J Biomater Tissue Eng.

[16] Marmarou A, Foda MA, van den Brink W, Campbell J, Kita H, Demetriadou K (1994). A new model of diffuse brain injury in rats. Part I: Pathophysiology and biomechanics. J Neurosurg.

[17] Wang YL, Lin CH, Chen CC, Chang CP, Lin KC, Su FC, Chou W (2019). Exercise preconditioning attenuates neurological injury by preserving old and newly formed HSP72-containing neurons in focal brain ischemia rats. Int J Med Sci.

[18] Faul M, Coronado V (2015). Epidemiology of traumatic brain injury. Handb Clin Neurol.

[19] Simon DW, McGeachy MJ, Bayır H, Clark RS, Loane DJ, Kochanek PM (2017). The far-reaching scope of neuroinflammation after traumatic brain injury. Nat Rev Neurol.

[20] Alluri H, Wiggins-Dohlvik K, Davis ML, Huang JH, Tharakan B (2015). Blood-brain barrier dysfunction following traumatic brain injury. Metab Brain Dis.

[21] Thal SC, Neuhaus W (2014). The blood-brain barrier as a target in traumatic brain injury treatment. Arch Med Res.

[22] Zarruk JG, Greenhalgh AD, David S (2018). Microglia and macrophages differ in their inflammatory profile after permanent brain ischemia. Exp Neurol.

[23] Li J, Li XY, Feng DF, Gu L (2011). Quantitative evaluation of microscopic injury with diffusion tensor imaging in a rat model of diffuse axonal injury. Eur J Neurosci.

[24] Jeong DU, Bae S, Macks C, Whitaker J, Lynn M, Webb K, Lee JS (2021). Hydrogel-mediated local delivery of dexamethasone reduces neuroinflammation after traumatic brain injury. Biomed Mater.

[25] Zhao ZL, Chen X, Zhu H (2013). Effects of glucocorticoids on traumatic brain injury related critical illness-related corticosteroid insufficiency. Chin Med J.

[26] Rowe RK, Harrison JL, Zhang H (2018). Novel TNF receptor-1 inhibitors identified as potential therapeutic candidates for traumatic brain injury. J Neuroinflammation.

